# Distinct Effects of Short Chain Fatty Acids on Host Energy Balance and Fuel Homeostasis With Focus on Route of Administration and Host Species

**DOI:** 10.3389/fnins.2021.755845

**Published:** 2021-10-22

**Authors:** Dehuang Kong, Lidewij Schipper, Gertjan van Dijk

**Affiliations:** ^1^Department of Behavioral Neuroscience, Groningen Institute for Evolutionary Life Sciences, University of Groningen, Groningen, Netherlands; ^2^Danone Nutricia Research, Utrecht, Netherlands

**Keywords:** gut microbiota, short chain fatty acids, gut-brain axis, host metabolism, energy homeostasis, metabolites

## Abstract

Accumulating evidence implicates gut-microbiota-derived metabolites as important regulators of host energy balance and fuel homeostasis, the underlying mechanisms are currently subject to intense research. In this review, the most important executors, short chain fatty acids, which both directly and indirectly fulfill the interactions between gut microbiota and host will be discussed. Distinct roles of individual short chain fatty acids and the different effects they exert on host metabolism have long been overlooked, which compromises the process of clarifying the sophisticated crosstalk between gut microbiota and its host. Moreover, recent findings suggest that exogenously administered short chain fatty acids affect host metabolism via different mechanisms depending on the routes they enter the host. Although these exogenous routes are often artificial, they may help to comprehend the roles of the short-chain-fatty-acid mechanisms and signaling sites, that would normally occur after intestinal absorption of short chain fatty acids. Cautions should be addressed of generalizing findings, since different results have appeared in different host species, which may imply a host species-specific response to short chain fatty acids.

## Introduction

The human body bears plenty of microbiota, including bacteria, viruses, archaea, and protists (Matijašić et al., 2020). The large intestine harbors the largest microbiota counts, up to 0.9 × 10^11^ bacteria cells g^–1^ of wet content (Gill et al., 2006; Sender et al., 2016). Moreover, the human gut microbiome contains nearly 10 million genes (Li et al., 2014), which is over 100 times more than the human genome (Eckburg et al., 2005). Gut microbiota has been recognized as an essential regulator of host cellular processes integral to several metabolic, physiological, and neuronal mechanisms that are vital to the host’s health (Fan and Pedersen, 2021). Disturbances in abundance and composition of gut microbiota may negatively impact these regulations and trigger disorders of energy balance and fuel homeostasis, such as obesity (Turnbaugh et al., 2009; Schwiertz et al., 2010) and type 2 diabetes (Wang et al., 2012). One mechanism by which gut microbiota can influence the host’s energy balance and fuel homeostatic state is via the production of microbial metabolites. Among them, short chain fatty acids (SCFAs) may be most important, of which acetate, propionate, and butyrate are the ones with highest concentrations (Ohira et al., 2017).

SCFAs in the cecum can be directly utilized by colonocytes as energy substrates or be involved in synthesis of carbohydrates and lipids of the host (den Besten et al., 2013). Furthermore, SCFAs also stimulate secretion of gut hormones or enter the circulatory system and target organs and tissues including the brain, where they can modulate control of energy balance and neuroendocrine/autonomic activity as well as behavior (Dalile et al., 2019). Specific SCFAs may, however, exert distinct effects in the host (Lin et al., 2012; den Besten et al., 2013; Jocken et al., 2018). For this reason, studies have been undertaken in which specific SCFAs have been exogenously delivered into humans and rodents. The mechanisms by which exogenous SCFAs influences host energy balance parameters may be different from the microbially produced SCFAs due to their varied routes of administration and sites of absorption (Koh et al., 2016; van der Beek et al., 2016; Li et al., 2018). The current review aims to shed light on the diverse pathways by which specific (exogenously applied) SCFAs may affect host physiological, metabolic, neural and behavioral mechanisms, in order to better understand the role of SCFA in regulating the host’s energy balance and fuel homeostasis.

## Synthesis of Short Chain Fatty Acids

Complex dietary fibers cannot be fully hydrolyzed by host enzymes in the small intestine of several species including humans and rodents, which renders their escape from degradation and absorption in the upper gastrointestinal tract. These indigestible carbohydrates serve as substrates to large intestinal anaerobic microbiota, resulting in production of SCFAs, predominantly acetate, propionate, and butyrate in colonic contents in an approximate molar ratio of 60:20:20 (Cummings et al., 1987; He et al., 2018; Ye et al., 2020).

Acetate is the most abundant SCFA in the gut in rodents and humans (Cummings et al., 1987; Ye et al., 2020). Gut microbiota generates acetate from dietary carbohydrates via acetyl-CoA, whereby pyruvate is produced by gut microbiota and then split into CO_2_ and acetyl-CoA. The resulting acetyl-CoA is converted to acetate. Acetate can also be produced via the Wood-Ljungdahl pathway of CO_2_ fixation through which CO_2_ is either reduced to formate via C_1_-body branch or CO via CO branch. Formate and CO can be further converted to acetyl-CoA by combining with a methyl group, the resulting acetyl-CoA will be converted to acetate (Ragsdale and Pierce, 2008). Propionate can be produced via three different pathways. The acrylate pathway begins with microbial conversion of pyruvate to lactate, which is subsequently converted to propionate. Furthermore, several bacteria have a specific preference for deoxy sugars (like fucose and rhamnose) to produce propionate via 1,2-propanediol through the propanediol pathway. Finally, propionate originating from hexose sugars is synthesized by utilizing methylmalonyl-CoA via the succinate pathway (Reichardt et al., 2014). Gut microbiota can produce butyrate via four pathways, the acetyl-CoA, glutarate, 4-aminobutyrate, and lysine pathways. All pathways merge at a central step, where crotonyl-CoA is transformed to butyryl-CoA, followed by butyrate synthesis either via butyryl-CoA:acetate CoA-transferase or via phosphotransbutyrylase and butyrate kinase (Louis and Flint, 2009; Vital et al., 2014).

## Specific Short Chain Fatty Acid-Producing Microbes

A wide range of enteric bacteria (including the highly prevalent *Akkermansia muciniphila* and *Blautia Hydrogenotrophica*) are capable of synthesizing acetate (Rey et al., 2010; Louis et al., 2014). Genomic and metagenomic analysis revealed comparatively more conserved bacterial genera for propionate and butyrate synthesis (Reichardt et al., 2014). The acrylate pathway is confined to the family Lachnospiraceae and Negativicutes. The propanediol pathway is also dominated by the Lachnospiraceae along with *Ruminococcus obeum* and *Roseburia inulinivorans*. The succinate pathway, which produces most of the propionate in the gut, mainly occurs in selective bacteria within the Bacteroidetes and Negativicutes (Reichardt et al., 2014). Recently, *Lawsonibacter asaccharolyticus* and *Intestinimonas butyriciproducens* were confirmed to be the novel butyrate-producing bacterial strains that encode the key enzymes for butyrate production (Bui et al., 2016; Sakamoto et al., 2018). In addition, Prevotellaceae, Clostridiaceae, and Lactobacillaceae were identified as potential butyrate producers (Esquivel-Elizondo et al., 2017).

## Distribution of Short Chain Fatty Acids in the Body and Effects in the Host

As mentioned earlier, SCFAs can be directly used in the gastrointestinal tract and/or can be absorbed and released into the bloodstream. The concentration of SCFAs is highest in the luminal contents of the cecum and the proximal colon. Comparison of SCFA concentrations in the colon, portal vein and hepatic vein revealed that butyrate is primarily consumed in gut epithelium while propionate is used in the liver (Cummings et al., 1987; Macfarlane et al., 1992). In line with this, butyrate was shown to be metabolized by mitochondria in colonocytes as energy source (Donohoe et al., 2011; Bourassa et al., 2016). In fact, energy provided by oxidation of SCFAs in colonocytes has been estimated to account for 60–70% of their total energy supply, with butyrate as its main source (Roediger, 1982; Clausen and Mortensen, 1995). Correspondingly, higher concentrations of butyrate-metabolizing enzymes have been found in colonocyte cultures *in vitro* compared to propionate-metabolizing enzymes (Kaiko et al., 2016). Energy homeostasis of colonocytes obtained from germ-free mice was restored to the same level as that of conventionally raised mice by either colonization with microbiota derived from conventionally raised mice, or with a butyrate-producing bacterial strain, supporting the notion that butyrate is preferentially used by colonocytes as energy source (Donohoe et al., 2011). Propionate, however, is primarily metabolized in the liver as gluconeogenic substrate (den Besten et al., 2013). Studies showed that propionate may participate in hepatic pyruvate cycling to regulate glucose homeostasis (Perry et al., 2016a) and may also ameliorate systematic inflammation (Chambers et al., 2019).

Recent work showed that the inferior mesenteric vein, which drains blood from the large intestine, bears the highest level of SCFAs, while the radial artery has the lowest SCFA concentrations. While a large portion of bacteria-derived SCFAs, especially butyrate and propionate, were shown to be consumed in the gut and the liver, an appreciable amount of acetate reached systemic circulation (Bloemen et al., 2009; Neis et al., 2019), which might mean that it affects other organs reached via transit of blood. Importantly, acetate is able to cross the blood-brain barrier and reaches central neural tissue, where it regulates appetite. Indeed, ^13^C-labeled acetate originating from fermentation in the digestive tract was able to reach hypothalamic neural circuits in control of food intake and energy balance (Frost et al., 2014).

The negligible systemic concentrations of bacteria-derived propionate and butyrate reaching other organs may not be sufficient to induce alteration of host metabolism (in contrast to exogenously supplemented SCFAs in higher concentrations). For example, human serum SCFAs concentrations were reported to be ∼100–150, ∼4–5, and ∼1–3 μM for acetate, propionate and butyrate, respectively (Wolever et al., 1997). The EC50 of the SCFA receptor GPR43 (i.e., an isoform of G protein couples receptors encoded by the fatty acid receptor gene 2; FFAR2) for acetate and propionate is ∼250–500 μM and the EC50 of GPR41 for propionate is ∼12–274 μM (Le Poul et al., 2003). Together, these works implicate that though most of the SCFAs are consumed in the gastrointestinal tract and liver, the remaining SCFAs (mostly acetate) can be transported via the bloodstream to target other organs and exert modulations on host metabolism, physiology and energy balance. Significant negative correlations between circulating (but not colonic) SCFAs were found with host cardiometabolic parameters in humans, but the causal mechanisms are difficult to disentangle (Müller et al., 2019), warranting the need for systematic investigating of the effects of exogenous applied specific SCFAs.

## Oral Applications of Short Chain Fatty Acids

Exogenous SCFAs application via oral route has frequently been studied, owing to its convenience, however, reaching different targets and probably in higher concentrations as compared to bacterially-produced SCFAs. In mice exposed to high fat diet, orally administered butyrate for 9 weeks decreased food intake (Li et al., 2018), while acetate administration for 6 weeks did not affect food intake (Kondo et al., 2009). In a direct comparison, dietary supplementation of butyrate and propionate in mice, but not acetate, indeed reduced food intake, despite the protective effects against diet-induced obesity and insulin resistance, which were observed in all treatment groups (Lin et al., 2012). De Vadder et al. showed that both orally-administered propionate and butyrate in rats exerted an effect on energy balance and fuel homeostasis by activating intestinal gluconeogenesis (IGN), however, dissimilar underlying mechanisms were driving them. Butyrate directly activated gene expression of IGN through a cAMP-dependent mechanism, while propionate *per se*, as a substrate of IGN, induced the activity of glucose-6-phosphatase and the expression of genes related to IGN via modulation of the FFAR3 (De Vadder et al., 2014). Furthermore, dietary propionate supplementation can also increase the expression of c-Fos (i.e., a marker for neuronal activity) in all areas of the dorsal vagal complex and the main hypothalamic regions, including the arcuate nucleus, perhaps pointing out the dependence of propionate-activated IGN on a gut-brain neural circuit, e.g., via afferent vagal fibers, and thus not necessarily involving direct access of propionate and butyrate to central neural tissue (De Vadder et al., 2014). Oral butyrate administration in high-fat-fed mice, however, resulted in quite the opposite effect, namely that it decreased the number of c-Fos-positive neurons within the arcuate nucleus (Li et al., 2018). Together with the inhibiting effect of oral butyrate administration on food intake, these results indicate an orexigenic (and potentially anabolic) neural circuit inhibited by the gut-brain axis involving butyrate, whilst propionate may stimulate anorexigenic (and potentially catabolic) pathways instead.

Interestingly, although acute oral propionate administration (in the form of inulin-propionate ester) in overweight adults also suppressed energy intake, this effect was observed together with increased levels of gut hormones peptide YY (PYY) and glucagon-like peptide-1 (GLP-1) secretion, which might indicated that propionate suppressed energy intake via stimulating gut hormone release that may tie in to brain pathways enhancing satiety and anorexia (Chambers et al., 2015).

## Non-Oral Applications of Short Chain Fatty Acids

As mentioned above, oral butyrate administration in mice decreased food intake by upregulating the protein expression of tyrosine hydroxylase and inhibiting the proportion of c-Fos positive (orexigenic) neurons in the hypothalamus that expressed neuropeptide Y (NPY), however, this effect was not noted with intravenous application of butyrate (Li et al., 2018). Anorexigenic effects of acetate were detected when acetate was either injected intraperitoneally into mice or after direct administration into the third ventricle of rats, suggesting that acetate may modulate food intake through direct impact on the central nervous system (Frost et al., 2014). Indeed, when intraperitoneally injected in the form of liposome-encapsulated acetate nanoparticles (which prevented acetate from entering the brain), peripheral acetate failed to induce appetite suppression (Frost et al., 2014; Sahuri-Arisoylu et al., 2016). Additionally, Frost et al. (2014) showed that profound up-, and down-regulation of, respectively, hypothalamic pro-opiomelanocortin (POMC) and hypothalamic agouti-related protein (AGRP) expression could be underlying these anorexigenic actions of acetate, as well as a reduction in the hypothalamic cellular energy sensor AMP kinase. It should be noted here that opposite effects of SCFAs on energy balance and fuel homeostasis have been reported too. For example, increased whole body acetate turn-over and plasma and higher fecal acetate concentrations were found to be causally related to hyperphagia and insulin resistance in high fat fed rats (Perry et al., 2016b). Bacterial cross feeding (Laverde Gomez et al., 2019) and the perturbations in gut microbiota induced by diet (Yang et al., 2019) may complicate the variation of SCFA profile in response to varied intestinal environment, potentially underlying contradictory observations and thus obscure our understanding of the host-microbiota interactions.

Human studies pointed out that distal, but not proximal, colonic acetate administration in obese individuals increased fat oxidation and fasting plasma PYY, which may be related to higher transfer of acetate to the circulation (van der Beek et al., 2016). SCFAs produced in the distal colon could be partly absorbed into the rectum and then drain into the inferior vena cava, thereby directly reaching the systemic circulation, circumventing uptake by liver (Canfora et al., 2015; van der Beek et al., 2016), and be taken into the arterial circulation potentially also reaching other tissues including the brain and adipose tissue. Along these lines, the adipocyte hormone leptin may also be implicated in the effects of SCFAs. Leptin, which circulates in the bloodstream roughly in proportion to the size of adipose tissue depots is an important regulator of energy balance by acting in aforementioned brain regions (Wing et al., 1996; Schwartz et al., 2000). Zaibi et al. showed that acetate, but not butyrate, increased mouse mesenteric adipocyte leptin secretion. Targeted deletion of GRP43 in mice caused loss of acetate-induced secretion of leptin in adipocytes derived from these knock-out mice, indicating that GPR43 is key in the SCFA-induced activation of leptin secretion (Zaibi et al., 2010). Similarly, GPR43 was shown to be highly expressed in four different adipose tissues, whereas GPR41 was not detected in adipose tissues (Hong et al., 2005; Kimura et al., 2011). A previous study reported that acetate and propionate were the most potent activators of GPR43 while butyrate was more active on GPR41 (Le Poul et al., 2003), which may partly explain the different effects of individual SCFAs on GPR41/43 activation and leptin secretion.

Jocken et al. demonstrated that specific SCFAs exerted distinct *in vitro* effects on lipolysis in human white adipocytes. When human multipotent adipose tissue-derived stem cells were incubated with mixtures of acetate, propionate, and butyrate or individual SCFAs, basal and the β-adrenergic receptor-mediated glycerol release were significantly suppressed in incubation with only acetate, while butyrate increased glycerol release and propionate did not affect their release. Additionally, the antilipolytic effect of acetate was found to be prevented when GPR41 and GPR43 in the adipocytes were inhibited, indicating an underlying mechanism mediated by these SCFA receptors involving GPR41 and GPR43 (Jocken et al., 2018). Taken together, these data suggest that acetate stimulates leptin production, but may prevent a local lipolytic effect of leptin at the site of its acetate-induced secretion.

## Discussion

**FIGURE 1:**
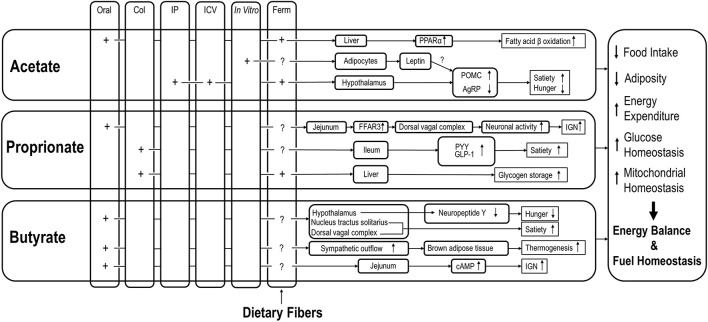
Effects of SCFAs via different routes of administration on various aspects of energy balance regulation and fuel homeostasis. COL, colonic; IP, intraperitoneal; ICV, intracerebroventricular; IGN, intestinal gluconeogenesis; Ferm, fermentation; A column of Ferm is added to point out the possibility that SCFA originating from dietary fibers may all have these effects as well, but those studies mostly lack the site-specific efficacy; Note that the figure did not summarize all the findings that were included in this manuscript since some contradictory findings may need further validation.

Overall, the reviewed data of distinct effects of specific SCFAs on host energy balance and fuel homeostasis suggest the following. Orally administered SCFAs may reach the small intestine and target local SCFA receptors, rather than being consumed by colonocytes. Intraperitoneally administered SCFAs may directly reach peripheral tissues and organs in supraphysiological concentrations and thus exert distinct impacts on host metabolism and physiology that probably are more profound compared to bacteria-derived SCFAs (see Table 1). In spite of different mechanisms and biochemical pathways, however, individual SCFAs may eventually lead to identical outcomes on host energy balance and fuel homeostasis. For example, propionate and butyrate may suppress energy intake via stimulation of anorexigenic neural pathway and inhibition of orexigenic neuron activity, respectively. The various mechanisms by which effects of SCFA, via various administration routes, on energy balance and fuel homeostasis are detailed in Figure 1.

**TABLE 1:** Summary of studies in short chain fatty acids (SCFAs) differentially affect host energy metabolism.

Host	SCFAs	Administered via	Dose	Duration	Main results	References
Mice mesenteric adipocytes	Acetate; butyrate	Incubation	Acetate (0.1 and 0.2 mM); Butyrate (0.2 mM)	–	• Acetate but not butyrate stimulated leptin secretion in wild-type mesenteric adipocytes	Zaibi et al., 2010
Human white adipocytes	Acetate; propionate; butyrate	Incubation	Ranging between 1 μmol/L and 1 mmol/L	–	• Acetate suppressed while butyrate increased basal and the β-adrenergic receptor-mediated glycerol release and propionate did not affect their release	Jocken et al., 2018
Mice	Acetate	Oral	0, 0.3, or 1.5% acetic acid at 10 mL/kg BW	6 weeks	• Acetate increased the expressions of genes for fatty-acid-oxidation- and thermogenesis-related proteins in the liver • No effect on FI	Kondo et al., 2009
Mice	Acetate; propionate; butyrate	Oral	Butyrate (5% w/w); propionate (4.3%); acetate (3.7%)	9 days	• Butyrate and propionate supplementation reduced FI • Acetate increased FI	Lin et al., 2012
Mice	Butyrate	Oral or IV injection	6 M, 0.15 mL for oral 15 mM or 150 mM, 0.1 mL for IV	9 weeks	• Oral rather than intravenous butyrate decreased FI and inhibited orexigenic neuron activity	Li et al., 2018
Mice or rats	Acetate	IP or ICV	IP: 500 mg/kg BW; ICV: 2.5 μmol	Acute	• Significant reduction in acute FI • Acetate may modulate FI through direct impact on the CNS	Frost et al., 2014
Rats	Propionate; butyrate	Oral	5% w/w	10–14 days	• Butyrate and propionate induced IGN via different mechanisms	De Vadder et al., 2014
Rats	Acetate	AT or ICV infusion	AT: 20 μmol kg^–1^min^–1^; ICV: 200 μM;	3 days or 4 weeks	• High fat diet induced acetate production • Increased acetate concentrations have been associated with obesity, insulin resistance	Perry et al., 2016b
Pigs	Acetate; propionate; butyrate	Dietary supplementation	0.1% in the diet	28 days	• Acetate reduced FI and body weight gain • SCFAs reduced lipogenesis, enhanced lipolysis via regulating related hormones and genes	Jiao et al., 2020
Humans	IPE	Dietary supplementation	10 g/day	Acute or 24 weeks	• Acute ingestion significantly reduced FI • 24 weeks supplementation reduced weight gain, intrahepatocellular lipid content	Chambers et al., 2015
Humans	Acetate	Distal or proximal colonic supplementation	100 or 180 mmol/l dissolved in 120 ml 0.9% NaCl	3 days	• Distal, but not proximal colonic acetate administration increased fat oxidation and fasting plasma PYY	van der Beek et al., 2016
Humans	IPE	Dietary supplementation	20 g/day	42 days	• IPE supplementation decreased proinflammatory interleukin-8 levels	Chambers et al., 2019

*AT, arterial; CNS, central nervous system; FI, food intake; ICV, intracerebroventricular; IGN, intestinal gluconeogenesis; IP, intraperitoneal; IPE, inulin-propionate ester; IV, intravenous; PYY, gut hormones peptide YY.*

An important aspect in consideration of these findings is host species. Energy balance and fuel homeostasis in response to SCFA may be dependent on species specific factors such as diet, anatomical and physiological characteristics of alimentary systems and SCFAs production capacity. Even comparison among rodent species may yield different outcomes. For instance, supplementation of propionate and butyrate to high-fat-fed mice, but not to high-fat-fed rats, reduced cumulative energy intake (Lin et al., 2012; De Vadder et al., 2014). Such incongruencies between mice and rats may be attributed to potential differences in gut microbiota signature, resulting in different physiological and metabolic states between hosts and their responses to SCFAs. Likewise, fecal SCFAs and lactate were shown to be different between mice and rats (Nagpal et al., 2018). Comparisons among rodent and human studies may be even more challenging. Firstly, the diversity and composition of gut microbiota and the level of SCFAs are more distinct between humans and rodents (Nagpal et al., 2018). Moreover, dissimilarities in physiological (and neural/endocrine) structure and function of the intestinal tract and the brain, and different dietary patterns and circadian rhythms between rodents and humans may also hinder the results of rodent experiments to be reproduced in humans (Frese et al., 2011; Thaiss et al., 2016). In addition, the scarcity of human data due to practical and ethical considerations brings about additional difficulties to compare experiments between rodent and human study in depth (Hamer et al., 2008; Blaak et al., 2020). Studies in animal species with neurobiological, metabolic and physiological systems more close to human (like pigs) may provide good alternatives (see e.g., Jiao et al., 2020). Future studies should also keep in mind these host-specific and site-specific direct and indirect mechanisms by which SCFAs can affect energy balance regulation and fuel homeostasis, highlighting their importance in health and development of diseases, that may in fact differ between hosts. Apart from proximal questions, there is finally also the need for unraveling the true natures of the signals provided by microbiota to the host, which will ultimately increase our understanding of the host-microbiota interplay.

## Author Contributions

DK wrote the first draft of the manuscript. DK, LS, and GD wrote sections of the manuscript. All authors contributed to manuscript revision, read, and approved the submitted version.

## Conflict of Interest

LS is employed by Danone Nutricia Research, Utrecht, Netherlands. The remaining authors declare that the research was conducted in the absence of any commercial or financial relationships that could be construed as a potential conflict of interest.

## Publisher’s Note

All claims expressed in this article are solely those of the authors and do not necessarily represent those of their affiliated organizations, or those of the publisher, the editors and the reviewers. Any product that may be evaluated in this article, or claim that may be made by its manufacturer, is not guaranteed or endorsed by the publisher.
